# ACGT newsletter (Farewell article)

**DOI:** 10.3332/ecancer.2010.ed8

**Published:** 2010-09-20

**Authors:** Jessica Michel Assoumou, Manolis Tsiknakis

**Affiliations:** 1 Administrative and Financial Coordinator, ERCIM, 2004, Route des Lucioles BP93, F-06902, Sophia Antipolis Cedex, France; 2 Scientific and Technical Coordinator, Foundation for Research & Technology-Hellas (FORTH), GR-71110 Heraklion, Greece

ACGT (Advancing Clinico-Genomic Trials on cancer: Open Grid Services for improving Medical Knowledge Discovery) is an Integrated Project (IP) funded by the 6th Framework Program of the European Commission under the Action Line “Integrated biomedical information for better health”.

Its high level objective has been the development of methods and systems for improved medical knowledge discovery and understanding through integration of biomedical information (e.g. using modeling, visualization, data mining and grid technologies). Biomedical data and information that have been considered include clinical information relating to tissues, organs or personal health-related information, but also information at the molecular and cellular level as acquired from genomics and proteomics research.

The project vision has been rooted in the realization that information arising from post-genomics research and genetic and clinical trials is rapidly providing the medical and scientific community with new insights, answers and capabilities when combined with advances in high-performance computing and informatics.

The over-arching objective of the ACGT project has been the provision of a unified technological infrastructure which facilitates the seamless and secure access and analysis of multi-level clinico-genomic data enriched with high-performing knowledge discovery operations and services.

During the course of the four and a half years of its life, the project has defined a detailed architectural blueprint and has developed, tested and validated a range of technologies, such as:

- new, domain-specific ontologies, built on established theoretical foundations and taking into account current initiatives, existing standard data representation models, and reference ontologies;
- innovative and powerful data exploitation tools, for example multi-scale modelling and simulation, considering and integrating from the molecular to the systems biology level, and from the organ to the living organism level;
- standards for exposing the properties of local sources in a federated environment;
- a biomedical grid infrastructure offering seamless mediation services for sharing data and data-processing methods and tools;
- advanced security tools including anonymisation and pseudonymisation of personal data according to European legal and ethical regulations;
- a Master Ontology on Cancer and use standard clinical and genomic ontologies and metadata for the semantic integration of heterogeneous databases;
- an ontology based Trial Builder for helping to easily set up new clinico-genomic trials, to collect clinical, research and administrative data, and to put researchers in the position to perform cross trial analysis; and
- data and literature mining services in order to support and improve complex knowledge discovery processes.

The technological infrastructure has been validated in a concrete setting of advanced clinical trials on cancer. Pilot trials have been selected based on the presence of clear research objectives, raising the need to integrate data at all levels of the human being. The project has targeted two major cancer diseases: breast cancer (BRCA) and paediatric nephroblastoma (PN).

In achieving these ambitious objectives, the project has brought together internationally recognised leaders in their respective fields, with the aim to deliver to the cancer research community an integrated Clinico-Genomic ICT environment enabled by a powerful grid infrastructure.

The ACGT consortium has pursued a long term vision to develop open-source, semantic and grid-based technologies in support of post genomic clinical trials in cancer research. Viewing the publications achieved over the life of ACGT we can say that lessons have been learned and good results were obtained.

Although the ACGT project is officially ending soon, the excellent research partnerships developed during the project will continue. The vision of becoming a pan-European voluntary network connecting individuals and institutions while enabling the sharing of data and tools and thus creating a European-wide web of cancer clinical research has been well advanced. The project has developed long lasting partnerships with some of the major stakeholders in the European Cancer Research arena, including ECCO (European Cancer Organisation), BIG (Breast International Group), SIOPE Europe (The European Society for Paediatric Oncology) and the European Clinical Research Infrastructures Network (ECRIN). Building upon the technologies, procedures and knowledge generated by the project, several ACGT partners – jointly with such important end user groups – are about to enter the second phase of implementation. This has been made possible through additional funding from the EU research programs (both HEALTH and ICT).

In parallel with these developments, the primary instruments for ACGT’s collaborative exploitation are well developed. The Center for Data Protection (CDP) has been established and is already actively engaged in service provision. Also the STaRC Initiative has grown into maturity. STaRC is intended to be a ‘Study, Trial and Research Centre’ that will exploit clinically relevant aspects of ACGT.

The concept behind STaRC has received significant recognition and support from patient organizations and patient support groups as well as from some regional governments. The activities for its official initiation are almost complete.

As the people that shared the coordination and management responsibilities of ACGT, we look back over the project’s lifetime remembering the many challenges that we faced and the many exchanges on very critical issues. Our conclusion is that the biggest strength and benefit of the ACGT project has been its people.

As you move on to new projects and new endeavors, we hope you will take with you fond memories of the achievements that you accomplished and the people that you worked with to accomplish them in the context of ACGT. We would like to thank the consortium members heartily for their hard work and enduring dedication to the project.

Many of us will meet one last time for the ACGT Final Review, to take place in Heraklion September 22–23. Until then, we wish you safe and happy summer holidays!

